# A Neural Network-Based Model for Predicting Saybolt Color of Petroleum Products

**DOI:** 10.3390/s22072796

**Published:** 2022-04-06

**Authors:** Nurliana Farhana Salehuddin, Madiah Binti Omar, Rosdiazli Ibrahim, Kishore Bingi

**Affiliations:** 1 Department of Chemical Engineering, Universiti Teknologi PETRONAS, Seri Iskandar 32610, Malaysia; lianafarhana@gmail.com (N.F.S.); madiah.omar@utp.edu.my (M.B.O.); 2 Department of Electrical and Electronics Engineering, Universiti Teknologi PETRONAS, Seri Iskandar 32610, Malaysia; rosdiazli@utp.edu.my; 3 School of Electrical Engineering, Vellore Institute of Technology, Vellore 632014, India

**Keywords:** automated prediction, multiple linear regression, Levenberg–Marquardt, scaled conjugate gradient, Saybolt color

## Abstract

Saybolt color is a standard measurement scale used to determine the quality of petroleum products and the appropriate refinement process. However, the current color measurement methods are mostly laboratory-based, thereby consuming much time and being costly. Hence, we designed an automated model based on an artificial neural network to predict Saybolt color. The network has been built with five input variables, density, kinematic viscosity, sulfur content, cetane index, and total acid number; and one output, i.e., Saybolt color. Two backpropagation algorithms with different transfer functions and neurons number were tested. Mean absolute error (MAE), root mean square error (RMSE), and coefficient of determination (R^2^) were used to assess the performance of the developed model. Additionally, the results of the ANN model are compared with the multiple linear regression (MLR). The results demonstrate that the ANN with the Levenberg–Marquart algorithm, tangent sigmoid transfer function, and three neurons achieved the highest performance (R^2^ = 0.995, MAE = 1.000, and RMSE = 1.658) in predicting the Saybolt color. The ANN model appeared to be superior to MLR (R^2^ = 0.830). Hence, this shows the potential of the ANN model as an effective method with which to predict Saybolt color in real time.

## 1. Introduction

Color measurement plays an essential role in the petroleum industry, as it aids in monitoring the product quality during storage and distribution [1]. Fuel that darkens in color indicates degradation or contamination [2]. Besides that, the quantifiable properties influence the selection of suitable refinement processes and final product classification. For instance, further processing is needed for natural gas liquids (NGLs) once removed from the natural gas or oil. Since the composition of NGLs can be vary depending on the gas field they are collected in, it is crucial to maintain their quality. Color is one of the quality indicators of NGLs’ quality. Slight changes in color may indicate contamination or degrading equipment. The low-quality NGLs can cause damage to refining equipment, necessitating costly repairs and clean-up [3]. Thus, constant monitoring by color measurement is needed to detect changes in quality. The measurement initially relies on manual visual inspection by an observer. However, this method is too subjective and may result in different interpretations of color expression [4]. Thus, two standards, namely, ASTM D1500 and Saybolt color (ASTM D156), have been developed by the American Society for Testing and Materials (ATSM) to determine the different ranges of petroleum products’ colors so that the results are reproducible and consistent [5,6,7]. Unlike ASTM D1500, which has a distinctive color scale, Saybolt is commonly used for slightly yellowish and highly refined petroleum products, not including low chroma products such as NGL, gasoline, kerosene, aviation fuels, naphtha pharmaceutical white oils, and petroleum waxes [7]. These types of oils are too light to be measured by ASTM D1500. The scale for Saybolt color ranges from −16, the darkest, to +30, the lightest.

Over the years, several methods have been proposed in the literature for color measurements, such as colorimeter usage [8,9], spectrophotometer usage [10,11] sensor-based methods [2,12], a color comparator method, and image analysis [5]. In most cases, samples need to be collected and analyzed in a laboratory before knowing the result. These procedures consume time, money, and energy. Hence, there have been several attempts to utilize automated predicted models, since they can measure in real-time conditions and are cost-effective [13,14]. Such a method expects the outcomes by analyzing historical and current data through machine learning and data mining. To date, the studies related to the automated predictive model for color measurement in the petroleum industry are still minimal. Regression modeling has been applied to develop correlations between the properties of petroleum products and Saybolt color. Surface tension, specific dispersion, refractive index [15], temperature [16], and API gravity [13] are among the properties that could cause variations in the colors of petroleum products. Khor et al. [13] were the first to demonstrate that mathematical correlations could be used to estimate petroleum color. Several multiple linear regressions with and without interactions were developed based on physical properties as predictors and the Saybolt color scale as the target. Although the results showed a statistically significant relationship, overfitting is a considerable problem in the models developed. Due to this, Leam et al. [14] explored different strategies that utilize stepwise regression techniques involving forward selection, backward elimination, and bidirectional elimination. Additionally, to achieve highly correlated models with higher adjusted coefficient of determination values, “regsubsets” and “glimuti” functions have been implemented in the study. Based on model validation, less than 5% prediction error for 75% of data samples has been achieved. Even though the regression models mentioned above perform well, they may have limitations. The non-linear and complex relationships between variables make obtaining high accuracy difficult. Aside from that, the models developed are still susceptible to overfitting when redundant regressors are present in the model.

In recent years, the applications of artificial neural networks (ANNs) for prediction have been steadily paving the way in the petroleum industries. The increasing number of studies on ANN applications has shown that the ANN poses significant advantages over classical regression due to its ability to mimic the human brain’s capabilities [17]. It quickly recognizes the data patterns and determines the closest findings to the actual values [18]. Compared to conventional modeling techniques, the ANN is widely used for complex systems that are difficult to approximate, particularly when the data are less than adequate [19]. Its high predictive capabilities can be attributed to the computing systems that simulate human brain processes called neurons [20]. The neuron acts as a single processor connected by weighted links [21]. Among the various types of ANNs, multilayer perceptron (MLP) with a backpropagation learning algorithm is the most widely used in solving problems. The network consists of one input layer, one hidden layer, and one output layer. The use of ANN is not new in the petroleum industry, since several studies have been reported. Agwu et al. [22] presented a full automation model to predict the downhole density of oil-based mud wells. Given the pressure and temperature changes during drilling, a complete understanding and accurate knowledge of drilling fluid density behavior are required. Such information can only be obtained through actual measurements using special equipment and slow analysis. However, the adaptation of a predictive method in such study has helped eliminate the need for equipment and concurrently provides more accurate measurements for the downhole mud density. Besides this, ANNs have been used to predict bottom-hole pressure [23], oil production [19], solution gas–oil ratio [24], flame temperature, and pollutant emissions [25] in the petroleum industry.

To the best of our knowledge, there is nearly no research in the scientific literature devoted to a study of automated color prediction based on ANN for petroleum products. The outcome of this work can benefit refineries or processing plants by:

- Saving valuable time spent deciding on the suitable refining feedstock that meets the requirements due to real-time measurements.
- Reducing the cost, since it eliminates sample handling and conditioning for the Saybolt color.
- Constantly monitoring product quality, which serves as an indicator of degrading equipment. This can help to prevent process efficiency losses.

Thus, the ANN model based on MLP was developed using different training algorithms, transfer functions, and several neurons to determine the best prediction model. The results from the ANN have been compared with the results from the multiple linear regression (MLR). All models were trained and tested to achieve high accuracy in prediction. This paper is organized as follows. Section 2 presents the proposed system models for prediction. Section 3 presents the results and discussion. Lastly, Section 4 provides the conclusion of the study.

## 2. Materials and Methods

**Figure 1:**
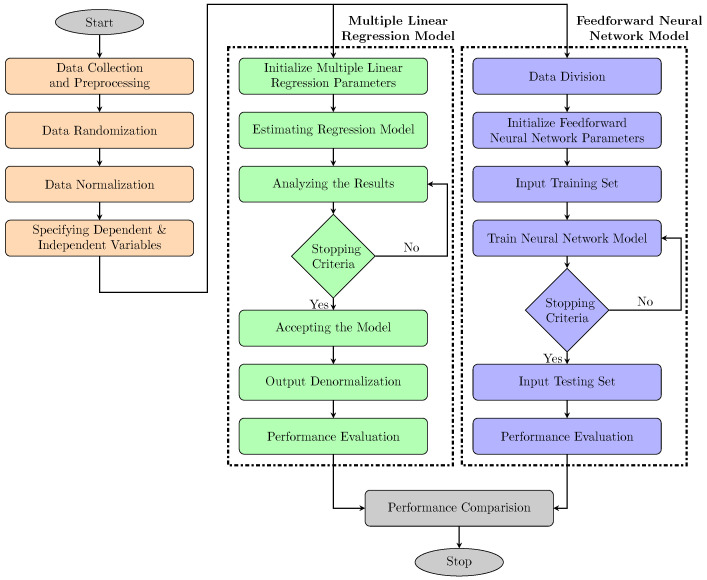
Modeling framework adopted in this work.

This section presents the methods used to develop the automated predictive model for Saybolt color. The historical data were trained using two different methods: An artificial neural network and multiple linear regression. The purpose was to get the most accurate Saybolt color prediction model. Linear regression was considered in this work due to its simplicity and extensive use in the past for similar problems. Meanwhile, the neural network was selected because of its increased recognition and superior performance in predicting non-linear relationships cases. The prediction model’s formation, either using ANN or MLR, can be divided into four main steps: Data collection and pre-processing, formation of the model, training, and testing the model’s performance. The overall flow design is shown in Figure 1, and each step is explained comprehensively in the following sub-sections.

### 2.1. Data Collection and Preprocessing

The dataset used in this study was obtained from assay reports. The data reported here were collected using various intelligent sensors for the product fractions (i.e., cuts) of condensates and light crude oils from Malaysian oil and gas fields. However, they are not provided here due to commercial confidentiality. Based on 19 assay reports, about five potential datasets with different numbers of independent variables were formed. The deletion method was used to remove missing or abnormal data. However, only one dataset with the highest number of independent variables was utilized in this work. This was to test as many of the variables that may influence the Saybolt color as possible. The data (*n* = 43) were first randomized into 35 training data and 8 test data. The parameters reported in this study are the basic properties measured for petroleum products and are commonly recorded in the assay reports. Density (D), kinematic viscosity at −20 °C (V), sulfur content (S), cetane index (C), and total acid number (A) are the input parameters we tested. The Saybolt color scale was the targeted output. Six combinations of the inputs were considered to determine the Saybolt color, as shown in Table 1. MATLAB version R2021a and 64-bit was used for developing the predictive models [26]. The neural net fitting toolbox from MATLAB was utilized to generate the code for the neural network. Then, the code was altered to test different architectures of neural networks. For linear regression, the “fitlm” function was used to build the regression model. All data were normalized by using the “mapstd” function in MATLAB before training and testing to yield zero means and unity standard deviation. This was due to different scales in the input parameters that consist of large and small magnitudes. The normalization process could reduce confusion of the learning algorithm on the importance of each parameter with a smaller magnitude that sometimes leads to rejection [27]. Scatter plots were plotted before training to analyze the relationships between variables.

### 2.2. Feedforward Neural Network-Based Model

**Figure 2:**
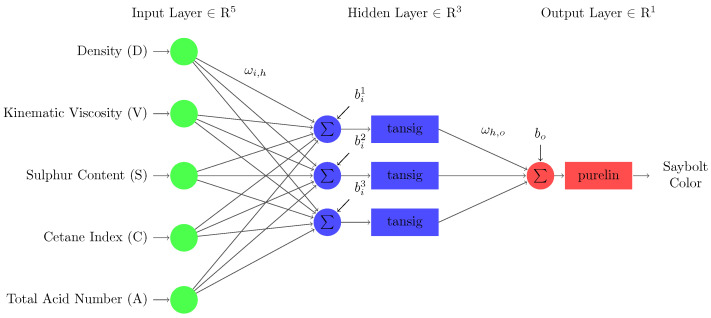
Feedforward neural network architecture constructed for this study.

A neural network is a modeling technique that simulates the neurons of a biological nervous system. Like the human brain, it learns from examples and utilizes them to solve a problem. Inputs, weights, transfer functions, and output are the essential components of the neuron [28]. Compared to regression-based models, the artificial neural network provides certain advantages, including its ability to deal with noisy and non-linear data. The network adopted in this work is represented in Figure 2. For example, “tansig” and “purelin” activation functions were used at hidden and output layers. A multilayer perceptrons model (MLP) with a single hidden layer was selected due to its easy implementation and ability as a universal approximator [29]. Typical MLP consists of an input layer that receives the initial values of the variables, a hidden layer to carry out operations, and an output layer to show the network results. The training of MLP entails determining the best weights of connections between neurons to achieve the slightest overall difference between the actual and predicted values of the dependent variable [30]. It works with the backpropagation (BP) algorithm in two stages. The first stage comprises a training set of input patterns spread from layer to layer by the neural network. Meanwhile, the second stage involves fine-tuning neural network weights based on the loss function calculated through the mean square error (MSE) method [20,31,32]. It is the sum of the squares of the differences between predicted and actual values. This causes the loss to be reduced and makes the model reliable by increasing its generalization.

### 2.3. Multiple Linear Regression-Based Model

Regression analysis is one of the most important statistical tools for determining the correlations between dependent (target) and independent (predictor) variables. The research is commonly initiated with linear regression because it is the most fundamental and comprehensive statistical and machine learning algorithm. In simple linear regression, the outcome of the dependent variable is predicted based on one independent variable, as in (1). Meanwhile, multiple linear regression (MLR) helps predict a relationship between the dependent variable and two or more independent variables [33]. In this case, since the inputs are more than one, MLR was used as a primary building for correlation by fitting a linear equation to the data. The MLR models were developed based on five different effects, namely, linear, interaction, pure quadratic, and quadratic, as given in (2)–(5), respectively [34,35].

(1) $$y=a_{0}+a_{1}x+\epsilon ,$$

(2) $$y=a_{0}+\sum_{i=1}^{m}a_{i}x_{i}+\epsilon ,$$

(3) $$y=a_{0}+\sum_{i=1}^{m}a_{i}x_{i}+\sum_{i,j=1;i<j}^{m}a_{ij}x_{i}x_{j},$$

(4) $$y=a_{0}+\sum_{i=1;p<3}^{m}a_{i}x_{i}^{p},$$

(5) $$y=a_{0}+\sum_{i=1}^{m,p<3}a_{i}x_{i}^{p}+\sum_{i,j=1;i<j;p,q<3}^{m}a_{ij}x_{i}^{p}x_{j}^{q}+\sum_{i,j=1;i\neq j;p,q<3;p<q}^{m}a_{ij}x_{i}^{p}x_{j}^{q},$$

where

- *y* is the predicted output;
- *x*, $x_{i}$, and $x_{j}$ are the independent input variables;
- $a_{i}$ and $a_{ij}$ are the regression coefficients;
- ϵ is the residual error.

### 2.4. Model Training and Testing

Model training was performed with various network configurations and learning parameters in order to find the model that produced the closest values to the actual Saybolt color values. The data division for training and testing for ANN and MLR was kept consistent throughout the study. This was done to compare their performances on similar grounds. Due to the small size dataset, overfitting tends to occur easily. Hence, the division ratio of training/testing is important. Empirical analysis has shown that allocating 20 to 30% of the original data for testing and the remaining data for training is the best division to avoid overfitting [36]. Thus, a preliminary study was conducted to determine the suitable ratio. The ratio of 80/20 (training/testing) gave better accuracy and less overfitting results. Further, the models were tested using a test dataset that was not used in training to ensure that the model could make accurate predictions and that the model was effectively trained. The training of the ANN models involved two types of BP algorithms for ANN models, namely, Levenberg–Marquardt (LM) and scaled conjugate gradient (SCG). Additionally, three different transfer functions, which are tangent sigmoid (tansig), logistic sigmoid (logsig), and radial basis function (radbas), were used to find the best ANN transfer function in the hidden layer. These functions have one thing in common: They all require the calculation of $e^{x}$. The equations of “tansig”, “logsig”, and “radbas” are given in (6)–(8). In all the equations, *x* is the independent variable and j=1,2,⋯,5. As for the output layer, the linear (purelin) transfer function was employed for all models. This function calculates a neuron’s output by simply returning the value passed to it [29,37].

(6) $$f\left(x\right)=\frac{e^{x}-e^{-x}}{e^{x}+e^{-x}},$$

(7) $$f\left(x\right)=\frac{1}{1+e^{-x}},$$

(8) $$f\left(x\right)=\exp(-\frac{1}{2\sigma_{j}^{2}}|x-x_{j}{|}^{2}).$$

**Figure 3:**
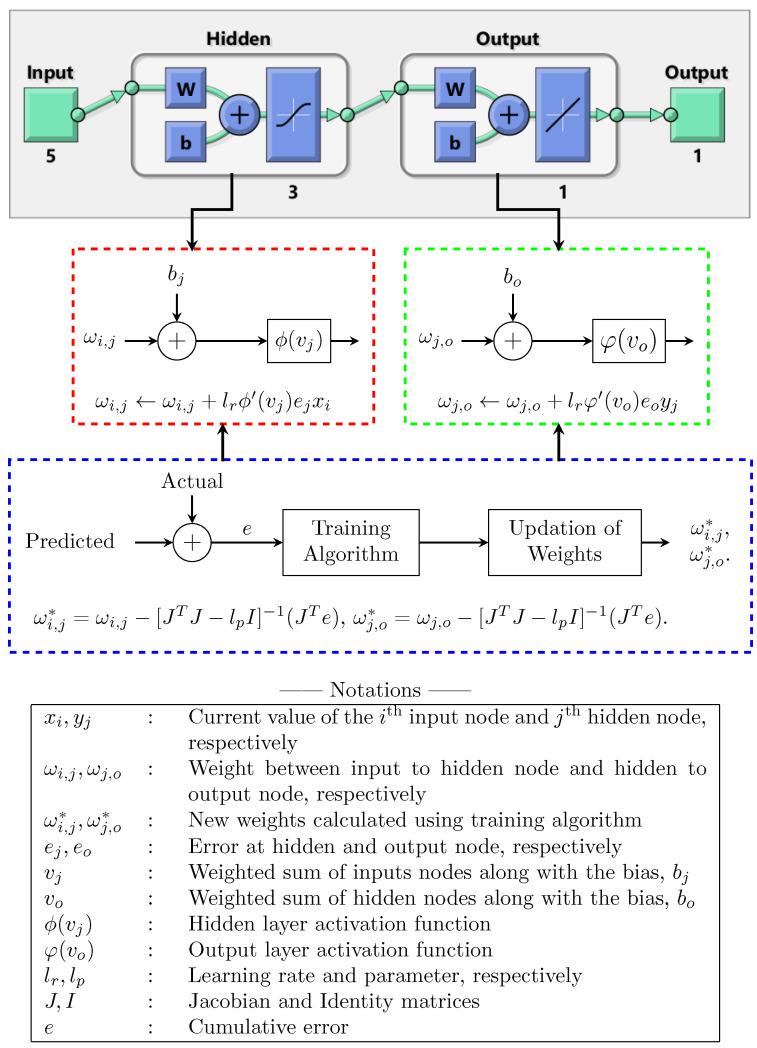
Adopted feedforward neural network model.

The feedforward neural network model adopted in this paper with a single hidden layer is shown in Figure 3. In the network model, the first and the last layers are the inputs (D, V, S, C, and A) and output (Saybolt Color) with five and one nodes, respectively. The middle layer is the hidden layer with three nodes. As an examplary case, the activation functions used at the hidden and output layers are, respectively, “tansig” and “purelin”. The mathematical formula of “tansig” and the delta rule to update weight, $\omega_{i,j}$, are shown in Figure 3’s red dotted box. Similarly, the mathematical formula of the “purelin” and the delta rule to update weight $\omega_{j,o}$ are shown in Figure 3’s green dotted box. The non-linear and linear combination of functions was used to achieve efficient training [38,39]. Further, Levenberg–Marquardt’s approach was employed to calculate the new weights $\omega_{i,j}^{*}$ and $\omega_{j,o}^{*}$ during the training, as shown in Figure 3. Weight update rules using the algorithm for $\omega_{i,j}^{*}$ and $\omega_{j,o}^{*}$ are also shown in Figure 3 (blue dotted box).

**Figure 4:**
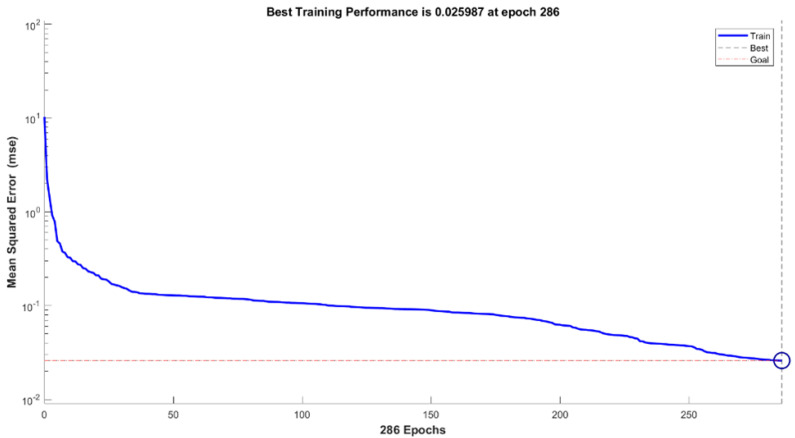
Error graph for the ANN models during training.

Each training iteration involves initial weights followed by output computation for all neurons, starting with the input layer. The number of iterations depends on the minimum value of the error function and stops when the error reaches sufficient values [20]. Default training parameters were employed for both LM and SCG—For instance, the number of epochs equal to 1000 and mean square error (MSE) as the performance function. Figure 4 presents one of the training error graphs of ANN models. The only set parameters were the performance goal (0.001) and minimum performance gradient $\left(1.0\times 10^{-5}\right)$. The values have been determined based on the average of the target data. Then, the training was iteratively performed in a loop of n=10 to obtain the best network for testing. The number of neurons is another parameter that was varied in this work. In a cause-and-effect prediction problem, the number of neurons in the input and output layer should correspond to the number of independent and dependent variables, respectively. There are, however, no rules to allow prior decisions to determine either the number of hidden layers or neurons [30]. Hence, to choose the suitable range of neuron numbers, a loop of neural networks with hidden neurons set from 1 to 10 was prerun using MATLAB before data training. The number of neurons chosen is critical because an insufficient number will cause difficulties in network learning, and an excessive number will result in unnecessary training time. On the other hand, the testing of MLR models involves the substitution of testing datasets into the equations obtained from training.

### 2.5. Model Performance Evaluation

The performances of ANN and MLR models in predicting Saybolt color were determined through mean absolute error (MAE), root mean square error (RMSE), and coefficient of determination (R^2^) as defined (9)–(11), respectively, as follows [40,41]:

(9) $$\mathrm{MAE}=\frac{1}{n_{s}}\sum_{k=1}^{n_{s}}\left(Y_{A,k}-Y_{P,k}\right),$$

(10) $$\mathrm{RMSE}=\sqrt{\frac{1}{n_{s}}\sum_{k=1}^{n_{s}}{\left(Y_{A,k}-Y_{P,k}\right)}^{2}},$$

(11) $$R^{2}=1-\frac{\sum_{k=1}^{n_{s}}{\left(Y_{A,k}-Y_{P,k}\right)}^{2}}{\sum_{k=1}^{n_{s}}{\left(Y_{A,k}-Y_{\mathrm{Avg}}\right)}^{2}},$$

where $Y_{A}$ and $Y_{P}$ are actual and predicted data, and $Y_{\mathrm{Avg}}$ is the average value of $Y_{A}$. MAE represents the average of total model error and assesses how close the prediction is to the actual values. Meanwhile, RMSE denotes the overall discrepancy between actual and predicted values [20,29]. Both parameters are preferred to be as small as possible to obtain the best performance by the prediction model. Additionally, the measurement of R^2^ is included in this work to show whether the actual and predicted values have a strong or weak relation. The closer the value of R^2^ to one, the higher percentage of data fit the proposed model [42].

## 3. Results and Discussion

**Figure 5:**
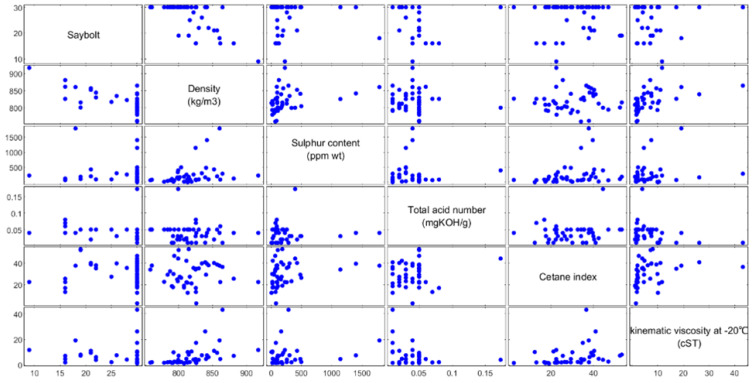
Scatterplots of Saybolt color versus five parameters: Density (D), kinematic viscosity at −20 °C (V), sulfur content (S), cetane index (C), and total acid number (A).

The relationships between all parameters were first investigated through scatter plots shown in Figure 5. No linear relationships could be observed between the target or Saybolt color and the predictors considered, namely, density, kinematic viscosity at −20 °C, sulfur content, cetane index, and total acid number. Additionally, the relations among predictors show no multicollinearity, a condition when two or more predictors are correlated, increasing the standard error of the coefficients. If this occurs, some variables can be statistically insignificant when significant [43]. Similar results were obtained by Khor et al. [13]. No linear relationship could be observed between the Saybolt color and physical properties of petroleum condensates, such as refractive index, kinematic viscosity at 40 °C, and characterization factor. The correlations between Saybolt color and input parameters were further investigated using ANN and MLR models. Their performances were evaluated through the values of MAE, RMSE, and R^2^.

### 3.1. Performances of ANN Models

In this study, a total of 43 data points were applied to design the ANN models for Saybolt color prediction. Two neural networks were built based on Levenberg–Marquart (LM) and scaled conjugate gradient (SCG) backpropagation algorithms with different transfer functions (tansig, logsig, and radbas) and different numbers of neurons in the hidden layer. This was done to find the best architecture for the ANN. Table 2 clearly shows that a network structure with an LM algorithm, tansig function, and three neurons outperformed others in prediction accuracy. Model 1 was the most successful in this structure, combining all five variables (density, kinematic viscosity at −20 °C, sulfur content, cetane index, and total acid number) as predictors. The model had the highest determination coefficient (R^2^ = 0.995), along with MAE and RMSE values of 1.000 and 1.658, respectively. The closer the value R^2^ to one, the higher the percentage of data that fit proposed model. It also indicates that the model can explain at least 0.99% of the measured data, supporting the applicability of using an ANN.

It is worth noting that increasing the number of neurons from two to three significantly improved the prediction accuracy of the ANN models. The MAE and RMSE values support this, as values decreased as neurons increased in number. However, more than three neurons resulted in overfitting problems in most trained models (data not shown). Overfitting is typically caused by excessive hidden neurons, which causes neural networks to overestimate the complexity of the target problem. It significantly reduces the generalization capability, resulting in a significant deviation in prediction [44]. Therefore, it is essential to determine the correct number of neurons to prevent the overfitting problem from occurring. Among the algorithms, the LM has shown better consistency with more hidden hidden neurons. Importantly, the accuracies of the model increased when neuron numbers increased from two to three. However, the accuracy of the SCG algorithm was not related to the number of hidden neurons.

Aside from that, the effects of different transfer functions were investigated. According to [45], although neural networks can learn any mapping, the inner ability to learn in a given case may necessitate flexible “brain modules” or appropriate transfer functions to solve the problem. For the LM algorithm, using logsig as a transfer function with three neurons led to a higher accuracy than using tansig and radbas. On the other hand, tansig showed better results, followed by logsig and radbas, in the SCG network. The performance values while using tansig and logsig as transfer functions. Both functions possess their own advantages: Tansig provides stronger gradients, hence reducing the chance of neuron saturation. It also prevents “biasing” of the gradients and causes a network with large connectivity to train faster when used with the backpropagation algorithm, as observed in [46]. Meanwhile, logsig is typically used in multilayer networks that are trained using BP algorithms, as it is differentiable [47]. The effects of input parameters on the Saybolt color were also investigated in this work. It is worth noting that model 1 comprised all variables for both algorithms, and all transfer functions tested recorded the highest accuracy in predicting the Saybolt color. Note that the performance of the ANN with different transfer functions varied with the changes in input. However, it could be observed that a prediction made by model 5 results in higher values of error and lower coefficients of determination for both algorithms. This indicates that the prediction accuracy is reduced in the absence of sulfur content as an input parameter.

Different algorithms, training functions, numbers of neurons, and hidden layers are typically manipulated when developing a neural network model to search for the best architecture that fits the data tested. A study in [48] also implemented a neural network to predict the color properties of polycarbonates. This was performed to achieve the correct color of plastic that is marketable. Pigment formulations and process parameters such as temperature, speed, and feed rate are the factors that can influence the color properties of the polymer. Five algorithms, including LM and SCG, were compared, and the number of neurons was varied. Statistical analysis, which included root mean square, the absolute fraction of variance, and mean square error, was used to evaluate the model’s performance. Similarly to Saybolt color prediction, SCG has shown good accuracy results in predicting the color of polycarbonates but is not consistent with the number of hidden neurons. Meanwhile, the LM algorithm has shown good accuracy performance and is consistent with a changing number of hidden neurons. The study demonstrated that the ANN’s performance would have been even better if a larger number of test runs had been performed. This would have provided a larger amount of experimental data for the network training.

Similarly to the present works, the number of data is the challenging factor in developing an ANN. A sample size determination in building machine learning is the most commonly debated issue, since it is subjected to the implemented application. However, this work highlights the ANN’s capabilities. High-performance results were achieved with the collected sample size in the Saybolt color prediction study. It contributes to creating a minimum base for the sample size to build an accurate Saybolt color prediction.

### 3.2. Performances of MLR Models

The ANN model’s performance has been compared with that of multiple linear regression. In this work, MLR was developed based on four different terms, namely, linear, interactions, pure quadratic, and quadratic, as shown in Table 3. It can be observed that regression through refined quadratic results in higher ranges of R^2^ and lower MAE, followed by quadratic, interaction, and linear trends. By including the pure quadratic terms in the models, the highest R^2^ value recorded was 0.83, and the lowest was 0.32. Model 2, which consists of four input variables (density, kinematic viscosity at −20 °C, sulfur content, and cetane index), has shown the best performance in predicting Saybolt color compared to others. It yields the lowest MAE, RMSE, and the highest coefficient. Equation (12) is the correlation produced by model 2.

(12) $$\mathrm{Saybolt}=0.4314-0.8967D+0.5124V+0.7238S-0.2673C-0.1191D^{2}-0.0232V^{2}-0.1980S^{2}-0.1107C^{2}.$$

Unlike the ANN, there were no clear relationships between the variables tested with the prediction accuracies for different effects. Overall, based on the coefficient of determination, which largely ranged from 0.50 to 0.70, it demonstrates that Saybolt color prediction through MLR models was less accurate. The high error and low correlations can be due to the incapability of MLR to understanding the non-linear relationship between petroleum properties and Saybolt color. The absence of linear relationships between Saybolt color and the properties of petroleum products was also observed in [14]. Thus, higher-order powers and interaction terms were explored to develop the correlations. The prediction models were designed up to a complexity of 24 input parameters using stepwise regression combined with statistical analysis. Nonetheless, the validation results showed a high percentage of deviation from the actual Saybolt color value. Hence, the higher-order of MLR is not investigated further in this work, as it may not be able to capture the complicated non-linear relationship.

### 3.3. Comparison between ANN and MLR

**Figure 6:**
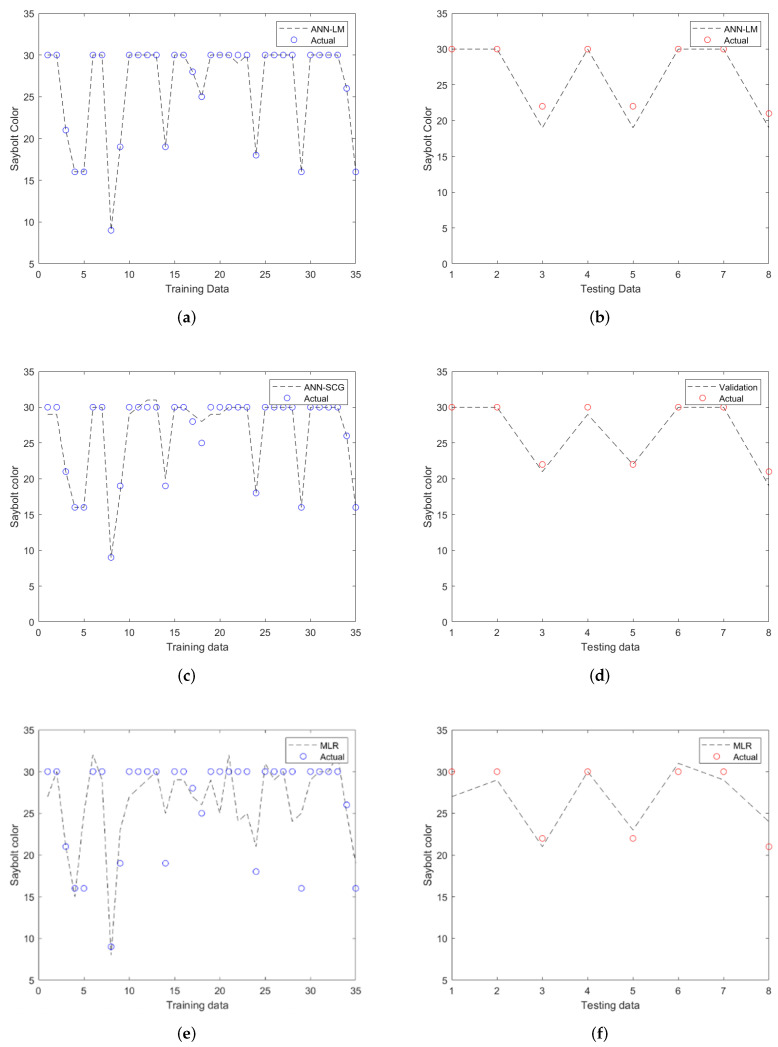
Comparison of actual and predicted Saybolt color of (**a**) training and (**b**) testing of ANN-LM, (**c**) training and (**d**) testing of ANN-SCG, (**e**) training, and (**f**) testing of MLR.

The performances of ANN models have been compared with that of the MLR model during both training and testing. In terms of values, it was apparent that the ANN-LM performance in predicting the Saybolt color was superior to the others due to its high R^2^ (0.994) and low MAE with RMSE values. This is supported by the results from Figure 6a that show the comparison of actual and predicted output values in training. From the plot, it can be seen that the ANN-LM model perfectly follows the actual results with R^2^ = 0.999. Thus, high prediction accuracy could be achieved when testing datasets have been fitted into the model (refer to Figure 6b). On the other hand, the plot of ANN-SCG also depicts high accuracy (refer to Figure 6c,d) during training and testing with R^2^ values of approximately 0.985 and 0.984, respectively. Unlike the others, the MLR model performed poorly during training (refer to Figure 6e). Despite this, the model was still acceptable because the R^2^ for testing data was more significant than 0.8 (refer to Figure 6f). The accuracy of the models developed in this study increased in the order of MLR > ANN-SCG > ANN-LM.

**Figure 7:**
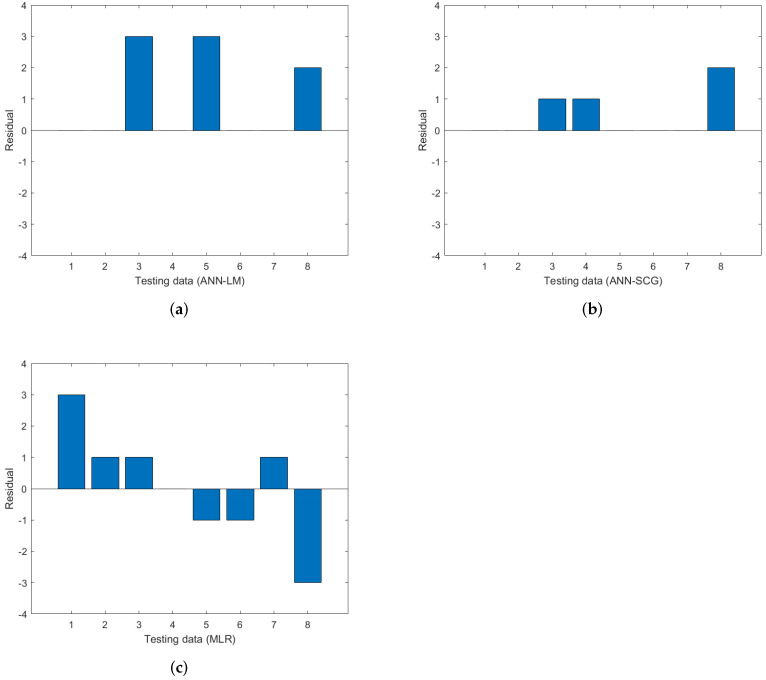
The residual comparison among (**a**) ANN-LM, (**b**) ANN-SCG and (**c**) MLR with the actual value of Saybolt color.

**Figure 8:**
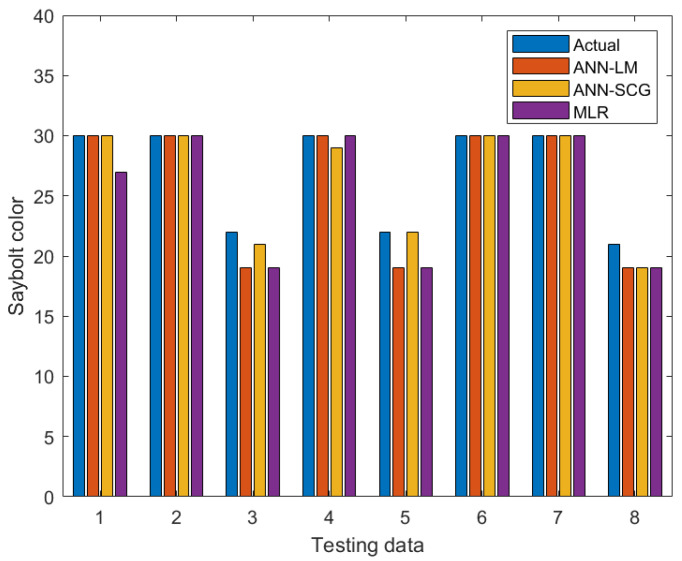
Comparison among ANN-LM, ANN-SCG, and MLR for predicting Saybolt value compared with next into the measured value in the laboratory.

Residuals between the actual and predicted data for each model were also calculated and plotted, as shown in Figure 7. The maximum residual for ANN-LM and MLR were determined to be around 3.0, whereas ANN-SCG was about 2.0 for the testing dataset. The results demonstrated ANN’s ability to provide high prediction accuracy compared to MLR. This is supported by the findings from Figure 8, in which the developed ANN results are closer and more accurate to the actual Saybolt color. These results show that the ANN method is a promising tool in predicting the Saybolt color.

The developed model has shown potential to be applied for monitoring and decision-making in the petroleum industry. Its use is not limited to specific regions because the inputs used are the basic properties tested for petroleum products and typically found in the assay reports. New testing data could be evaluated using the prediction model as long as the independent variables with similar units are used. Further, even though the current neural network was trained with a small dataset, it can always be updated by introducing additional data to improve performance. Retraining a new sample set of data is also needed if the inputs and output are outside the boundaries. Through MATLAB, the current weights can be obtained and assigned to the corresponding position in the new network. Network training will then involve a data combination of the previous and latest. The former dataset needs to be included by representing it as a subset and added to the new data to preserve the dominant characteristics of the first dataset. Meanwhile, the new dataset could be saved in the workspace and loaded into the new network for retraining.

In terms of applications, it is known that detecting color changes in petroleum products is the simplest, most rapid determination method for detecting suitable refining processes or monitoring product quality. For example, the developed ANN model could be utilized to save valuable time in deciding to purchase a condensate as a refining feedstock. Conventionally, samples are taken to measure the Saybolt color scale using the equipment. This method consumes time and is costly. However, with the developed method, accurate prediction of the Saybolt value can be performed by inputting the condensate properties, which can be found in the assay report. If the quality does not meet the threshold, no further condensate analysis is required. Hence, the method reduces the number of samples to be tested, since only quality products that are being considered. To implement the proposed model in a real-working environment and make it assessable, an application can be developed with in-house technology so that the model is flexible for modification and enhancement. This study contributes to the exploration of artificial intelligence as prediction models for the Saybolt color. It could provide context and guidance to researchers seeking a method to relate the non-linear relationship in color measurement or sensor development for quality control.

## 4. Conclusions

This work focused on predicting the Saybolt color, which plays a crucial role in monitoring the quality and determining the following process for petroleum products. To develop an ANN model with high accuracy in predicting Saybolt color, density, kinematic viscosity at −20 °C, sulfur content, cetane index, and total acid number were used as input parameters, and Saybolt color as the output. Different learning methods, transfer functions, and neuron numbers were tested to obtain the most suitable prediction model. A model developed with all five parameters, the Levenberg–Marquardt backpropagation algorithm and tangent sigmoid with three neurons, was the most accurate model for predicting the Saybolt value. This model had the highest R^2^ (0.995), and lower values of MAE (1.000) and RMSE (1.658) compared to others. The comparison between the ANN model with multiple linear regression showed that the ANN is superior in providing an accurate value of Saybolt color and can be used as an alternative to conventional measurement methods. Implementing this model in measuring Saybolt color could benefit refineries or processing plants with real-time measurement and reduce the dependency on the equipment, besides being cost-effective, as the sampling number is reduced. For future study, it would be worth investigating the influences of other properties of petroleum products on the Saybolt color. Additionally, by identifying the strongest and weakest independent variables that affect the value of the Saybolt color, a model with minimum inputs could be developed and optimized.

## Figures and Tables

**Table 1:** Combinations of input parameters.

Model	Name	Inputs
1	DVSCA	Density (kg/m^3^) Kinematic viscosity at −20 °C (cST) Sulfur content (ppm wt) Cetane index Total acid number (mgKOH/g)
2	DVSC	Density (kg/m^3^) Kinematic viscosity at −20 °C (cST) Sulfur content (ppm wt) Cetane index
3	DVSA	Density (kg/m^3^) Kinematic viscosity at −20 °C (cST) Sulfur content (ppm wt) Total acid number (mgKOH/g)
4	DSCA	Density (kg/m^3^) Sulfur content (ppm wt) Cetane index Total acid number (mgKOH/g)
5	DVCA	Density (kg/m^3^) Kinematic viscosity at −20 °C (cST) Cetane index Total acid number (mgKOH/g)
6	VSCA	Kinematic viscosity at −20 °C (cST) Sulfur content (ppm wt) Cetane index Total acid number (mgKOH/g)

**Table 2:** Performances of ANN models during testing.

Number of Neurons			2			3		
Training Function	Model	Transfer Function	MAE	RMSE	R^2^	MAE	RMSE	R^2^
Levenberg–Marquardt	1	tansig	0.750	1.500	0.867	1.750	2.693	0.993
	2		2.625	5.601	0.786	1.750	3.082	0.957
	3		1.750	2.236	0.759	1.250	2.000	0.825
	4		2.125	3.446	0.557	1.000	1.803	0.975
	5		1.875	2.716	0.597	2.625	3.021	0.612
	6		1.500	2.500	0.766	1.250	1.658	0.833
	1	logsig	1.750	3.082	0.957	1.000	1.658	0.995
	2		2.750	3.279	0.732	2.750	3.082	0.760
	3		1.750	2.236	0.759	1.875	3.182	0.974
	4		1.375	3.221	0.484	1.250	2.550	0.634
	5		2.125	3.062	0.572	26.875	45.622	0.851
	6		1.500	2.500	0.766	1.250	1.658	0.833
	1	radbas	1.625	2.208	0.727	1.250	1.658	0.886
	2		1.500	1.732	0.838	1.625	2.372	0.812
	3		1.750	2.345	0.758	1.500	2.000	0.779
	4		1.875	2.622	0.753	1.500	2.345	0.725
	5		1.750	2.179	0.780	1.750	2.449	0.695
	6		2.000	2.121	0.732	3.375	4.430	0.755
Scaled Conjugate Gradient	1	tansig	0.750	1.500	0.867	0.500	0.866	0.984
	2		2.125	3.221	0.615	4.375	7.706	0.875
	3		1.750	2.449	0.650	1.125	2.031	0.917
	4		0.750	2.121	0.760	2.000	2.828	0.795
	5		2.250	3.606	0.462	2.375	3.824	0.559
	6		1.750	2.550	0.618	1.500	2.121	0.843
	1	logsig	1.250	1.803	0.842	1.000	1.500	0.927
	2		2.125	3.062	0.712	1.750	2.500	0.786
	3		1.750	2.236	0.759	1.125	2.031	0.917
	4		0.750	2.121	0.760	1.375	2.716	0.627
	5		2.875	3.657	0.501	2.500	3.969	0.633
	6		1.500	2.500	0.766	1.500	2.121	0.843
	1	radbas	1.875	2.622	0.753	1.125	1.275	0.904
	2		1.500	1.802	0.817	1.625	2.031	0.791
	3		1.750	2.236	0.759	1.875	3.182	0.899
	4		1.875	2.622	0.753	1.750	3.122	0.626
	5		1.750	2.121	0.803	1.750	2.550	0.673
	6		1.750	2.345	0.817	1.750	2.291	0.762

**Table 3:** Performances of MLR models during testing.

Model	Type	MAE	RMSE	R^2^
1	Linear	2.375	3.142	0.564
2		2.500	3.240	0.573
3		2.375	3.142	0.518
4		2.000	2.550	0.616
5		2.250	3.041	0.572
6		3.750	4.000	0.024
1	Interaction	2.750	4.062	0.279
2		2.000	2.646	0.588
3		2.125	2.669	0.666
4		2.000	2.915	0.536
5		3.000	4.387	0.108
6		2.125	2.574	0.604
1	Pure quadratic	2.000	2.236	0.699
2		1.375	1.696	0.830
3		2.250	2.449	0.651
4		1.625	2.150	0.721
5		2.250	2.598	0.592
6		3.250	3.571	0.318
1	Quadratic	2.125	2.622	0.696
2		2.000	2.550	0.607
3		1.500	2.000	0.757
4		1.625	2.092	0.745
5		2.500	3.969	0.254
6		1.750	2.121	0.729

## Data Availability

Data available in a publicly accessible repository.
